# Sublineage structure analysis of *Mycobacterium tuberculosis* complex strains using multiple-biomarker tensors

**DOI:** 10.1186/1471-2164-12-S2-S1

**Published:** 2011-07-27

**Authors:** Cagri Ozcaglar, Amina Shabbeer, Scott Vandenberg, Bülent Yener, Kristin P Bennett

**Affiliations:** 1 Computer Science Department, Rensselaer Polytechnic Institute, Troy, NY, USA; 2 Mathematical Sciences Department, Rensselaer Polytechnic Institute, Troy, NY, USA; 3 Computer Science Department, Siena College, Loudonville, NY, USA

## Abstract

**Background:**

Strains of *Mycobacterium tuberculosis* complex (MTBC) can be classified into major lineages based on their genotype. Further subdivision of major lineages into sublineages requires multiple biomarkers along with methods to combine and analyze multiple sources of information in one unsupervised learning model. Typically, spacer oligonucleotide type (spoligotype) and mycobacterial interspersed repetitive units (MIRU) are used for TB genotyping and surveillance. Here, we examine the sublineage structure of MTBC strains with multiple biomarkers simultaneously, by employing a tensor clustering framework (TCF) on multiple-biomarker tensors.

**Results:**

Simultaneous analysis of the spoligotype and MIRU type of strains using TCF on multiple-biomarker tensors leads to coherent sublineages of major lineages with clear and distinctive spoligotype and MIRU signatures. Comparison of tensor sublineages with SpolDB4 families either supports tensor sublineages, or suggests subdivision or merging of SpolDB4 families. High prediction accuracy of major lineage classification with supervised tensor learning on multiple-biomarker tensors validates our unsupervised analysis of sublineages on multiple-biomarker tensors.

**Conclusions:**

TCF on multiple-biomarker tensors achieves simultaneous analysis of multiple biomarkers and suggest a new putative sublineage structure for each major lineage. Analysis of multiple-biomarker tensors gives insight into the sublineage structure of MTBC at the genomic level.

## Background

Tuberculosis (TB), a bacterial disease caused by *Mycobacterium tuberculosis* complex (MTBC), is a leading cause of death worldwide. In the United States, isolates from all TB patients are routinely genotyped by multiple biomarkers. The biomarkers include Spacer Oligonucleotide Types (spoligotypes), Mycobacterial Interspersed Repetitive Units - Variable Number Tandem Repeats (MIRU-VNTR), IS6110 Restriction Fragment Length Polymorphisms (RFLP), Long Sequence Polymorphisms (LSPs), and Single Nucleotide Polymorphisms (SNPs).

Genotyping of MTBC is used to identify and distinguish MTBC into distinct lineages and/or sublineages that are quite useful for TB tracking, TB control, and examining host-pathogen relationships [1]. The six main major lineages of MTBC are *M. africanum*, *M. bovis*, *M. tuberculosis* subgroup Indo-Oceanic, *M. tuberculosis* subgroup Euro-American, *M. tuberculosis* subgroup East Asian (Beijing) and *M. tuberculosis* subgroup East-African Indian (CAS). Other major lineages exist such as *M. canettii* and *M. microti*, but they do not commonly occur in the US, so we do not consider them here. These major lineages can be definitively characterized using LSPs [2], but typically only spoligotypes and MIRU are collected for the purpose of TB surveillance. Classification, similarity search, and expert-rule based methods have been developed to correctly map isolates genotyped using MIRU and/or spoligotypes to the major lineages [3-5].

While sublineages of MTBC are routinely used in the TB literature, their exact definitions, names, and numbers have not been clearly established. The SpolDB4 database contains 39,295 strains and their spoligotypes with the vast majority of them labeled and classified into 62 sublineages [6], but many of these are considered to be “potentially phylogeographically-specific MTBC genotype families”, rather than distinct phylogenetic sublineages with known biomarkers. Therefore, further analysis is needed to confirm these sublineages. The highly-curated MIRU-VNTR*plus* website, which focuses primarily on MIRU, defines 22 sublineages. New definitions of sublineages based on LSPs and SNPs are being discovered; e.g. the RD724 polymorphism corresponds to the previously defined SpolDB4 T2 sublineage, also known as the Uganda strain in MIRU-VNTR*plus*[7]. Now large databases using spoligotype, MIRU patterns, and RFLP exist. The United States Centers for Disease Control and Prevention (CDC) has gathered spoligotypes and MIRU isolates for over 37,000 patients. Well-defined TB sublineages based on spoligotype and MIRU are critical for both TB control and TB research.

The goal of this paper is to examine the sublineage structure of MTBC on the basis of multiple biomarkers. The proposed method reveals structure not captured in SpolDB4 spoligotype families because SpolDB4 sublineage only take into account a single biomarker, spoligotypes. A spoligotype-only tool, SPOTCLUST, was used to find MTBC sublineages using an unsupervised probabilistic model, reflecting spoligotype evolution [8]. A key issue is to combine spoligotype and MIRU into a single unsupervised learning model. When MIRU patterns are considered, SpolDB4 families that are well-supported by spoligotype signatures may become ambiguous, or allow subdivision/merging of the families. Existing phylogenetic methods can be readily applied to MIRU patterns, but specialized methods are needed to accurately capture how spoligotypes evolve. It is not known how to best combine spoligotype and MIRU patterns to infer a phylogeny. The online tool www.MIRUVNTRplus.org determines lineages by using similarity search to a labeled database. The user must select the distance measure which is defined using spoligotypes and/or MIRU patterns, possibly yielding different results.

In this study, we develop a tensor clustering framework to find the sublineage structure of MTBC strains labeled by major lineages based on multiple biomarkers. This is an unsupervised learning problem. We generate multiple-biomarker tensors of MTBC strains for each major lineage and apply multiway models for dimensionality reduction. The model accurately captures spoligotype evolutionary dynamics using contiguous deletions of spacers. The tensor transforms spoligotypes and MIRU into a new representation, where traditional clustering methods apply without users having to decide a *priori* how to combine spoligotype and MIRU patterns. Strains are clustered based on the transformed data without using any information from SpolDB4 families. Clustering results lead to the subdivision of major lineages of MTBC into groups with clear and distinguishable spoligotype and MIRU signatures. Comparison of the tensor sublineages with SpolDB4 families suggests dividing or merging some SpolDB4 families. As a way of validating multiple-biomarker tensors, we use them in a supervised learning model to predict major lineages using spoligotype deletions and MIRU. We compare the prediction accuracy of the multiple-biomarker tensor model created with N-PLS (N-way partial least squares) with the 2-way PLS applied to matrix data and an existing conformal Bayesian Network approach.

In the next section, we give a brief background on clustering and multiway analysis of post-genomic data, spoligotyping, and MIRU typing.

### Clustering post-genomic data

Data clustering is a class of techniques for unsupervised classification of data samples into groups of similar behavior, function, or trait [9]. Clustering can be used in post-genomic data analysis to group strains with similar traits. It is common practice to use different clustering methods and use a *priori* biological knowledge to interpret the clusters, but computational cluster validation is needed to validate results without prior knowledge for unsupervised classification. A great survey by Handl et al. outlines the steps of computational cluster analysis on post-genomic data [10]. An application of computational cluster validation on microarray data by Giancarlo et al. compares the results of clusterings using various cluster validation indices [11]. Eisen et al. clusters gene expression data which groups genes of similar functions [12]. Improved clustering techniques have been developed, but how to combine multiple sources of information in one clustering is an open question.

### Application of multiway models to post-genomic data clustering

Clustering on post-genomic data can be accomplished based on multiple sources of ground truth. The ground truth can be based on multiple biomarkers, host and pathogen, or antigen and antibody. A survey by Kriegel et al. outlines the methods for finding clusters in high-dimensional data [13]. Analysis of multiway arrays for data mining is frequently used today in various fields, including bioinformatics, to use multiple sources of prior information simultaneously [14]. Alter and Golub use higher-order eigenvalue decomposition on a *networks* × *genes* × *genes* tensor and find significant subnetworks associated with independent pathways in a genome-scale network of relations among all genes of cellular systems [15]. Omberg et al. use higher-order singular value decomposition on DNA microarray data, obtaining the core tensor of *eigenarrays* × *x-eigengenes* × *y-eigengenes* and finding correlation between genomes in the subtensors of the core tensor [16]. Multiway analysis of EEG data identifies epileptic seizures [17]. Use of common partitive and hierarchical clustering algorithms accompanied with multiway modeling of high-dimensional data finds functionally related genes in stem cells [18]. Similarly, multiple biomarkers of the MTBC genome can be used to cluster MTBC strains.

### Spoligotyping

Spoligotyping is a DNA fingerprinting method that exploits the polymorphisms in the direct repeat (DR) region of the MTBC genome. The DR region is a polymorphic locus in the genome of MTBC which consists of direct repeats (36 bp), separated by unique spacer sequences of 36 to 41 bp [19]. The method uses 43 spacers, thus a spoligotype is typically represented by a 43-bit binary sequence. Zeros and ones in the sequence correspond to the absence and presence of spacers respectively. Mutations in the DR region involve deletion of one or more contiguous spacers. To capture this mechanism of mutation in our model, we find informative contiguous spacer deletions and represent spoligotype deletions as a binary vector, where one indicates that a specific contiguous deletion occurs (i.e. a specified contiguous set of spacers are all absent) and zero means at least one spacer is present in that contiguous set of spacers.

Large datasets of MTBC strains genotyped by spoligotype have been amassed such as SpolDB4 [6] and a more extended online version SITVIT (http://www.pasteur-guadeloupe.fr:8081/SITVITDemo/index.jsp). Spoligotypes can be readily used to identify commonly accepted major lineages of MTBC with high accuracy [4]. SpolDB4 defined a set of phylogeographic sublineages or families based on expert derived rules that are in common use in the TB community. In contrast to the major lineages that have been validated by more definitive markers such as single nucleotide polymorphisms and long sequence polymorphism, the exact definition of MTBC sublineages and the accuracy of the SpolDB4 families created only using spoligotypes remain open questions.

### MIRU-VNTR typing

MIRU is a homologous 46-100 bp DNA sequence dispersed within intergenic regions of MTBC, often as tandem repeats. MIRU-VNTR typing is based on the number of tandem repeats of MIRUs at certain identified loci. Among these 41 identified mini-satellite regions on the MTBC genome, different subsets of sizes 12, 15, and 24 are proposed for the standardization of MIRU-VNTR typing [3]. In this study, we use 12 MIRU loci for genotyping MTBC. Thus, the MIRU pattern is represented as a vector of length 12, each entry representing the number of repeats in each MIRU locus.

## Results

We used the tensor clustering framework to cluster MTBC strains using multiple biomarkers, and compared the clustering to SpolDB4 sublineages. Next, we used supervised tensor learning and classified MTBC strains into major lineages using spoligoype deletions and MIRU patterns. We compared multiway and two-way supervised learning methods based on their prediction accuracy for major lineage classification. In the following section, we introduce multiple-biomarker tensors and present unsupervised and supervised learning experiments on multiple-biomarker tensors.

### Multiple-biomarker tensor analysis of strain data

Multiple biomarkers of the MTBC genome in a relational database can be represented as a high-dimensional dataset for multiway analysis. The multiple-biomarker tensor is constructed this way, with one of the modes representing strains and other modes representing biomarkers. In our experiments, we use this multidimensional array or tensor with three modes representing strains, spoligotype deletions, and MIRU patterns. This multiple-biomarker tensor captures three key properties of MTBC strains: spoligotype deletions, number of repeats in MIRU loci, and coexistence of spoligotype deletions with MIRU loci.

**Figure 1:**
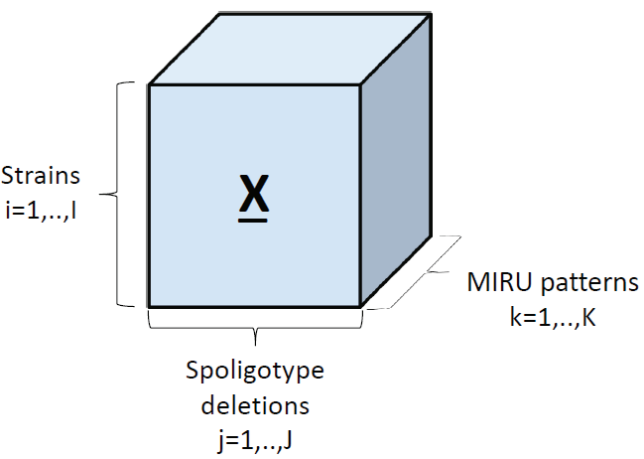
**Multiple-biomarker tensor*** Strains* x *Spoligotype deletions* x *MIRU patterns* tensor. Each entry X(*i*, *j*, *k*) of the tensor represents the number of repeats in MIRU locus *k* of strain *i* with spoligotype deletion *j.*

**Figure 2:**
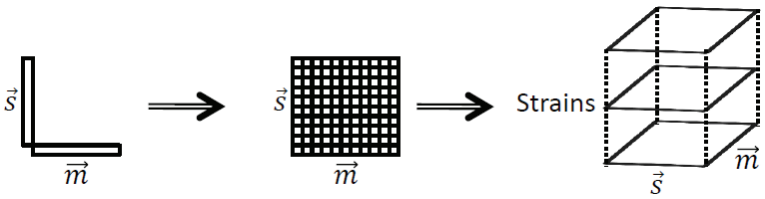
**Generation of multiple-biomarker tensor** Biomarker kernel matrix  for each strain forms multiple-biomarker tensor. Vector  represents spoligotype deletions and  represents MIRU patterns.

The strain dataset is arranged as a three-way array with strains in the first mode, spoligotype deletions in the second mode, and MIRU patterns in the third mode. Each entry **X**(*i*, *j*, *k*) in the tensor corresponds to the number of repeats in MIRU locus *k* of strain *i* with spoligotype deletion *j.* If spoligotype deletion *j* does not exist in strain *i*, then the tensor entry **X**(*i*,*j*,.) is 0. Thus, strain datasets are formed as *Strains* × *Spoligotype deletions* × *MIRU patterns* tensors, as shown in Figure 1. Mathematically, each strain is represented as the outer product of the binary spoligotype deletion vector and the MIRU pattern vector, which results in a biomarker kernel matrix. Biomarker kernel matrices of the same size for each strain form the multiple-biomarker tensor. Generation of the multiple-biomarker tensor from biomarkers of each strain is shown in Figure 2. We represent spoligotype deletions with a binary vector , where *s_i_* ∈ {0,1}, *i* ∈ {1,‥,*n*}, and *n* is the number of informative spoligotype deletions found using the feature selection algorithm, detailed in the methods section. We represent 12-loci MIRU with a digit vector , where *m_j_* ∈ {1,‥,9, > 9} and *j* ∈ {1,‥,12}. The entries of the multiple-biomarker tensor which combines spoligotype and MIRU information can be formulated as:

where

and r_ik_ is the number of repeats in MIRU locus k of strain i. Multiple-biomarker tensors can be used for both unsupervised and supervised learning. Next, we use the unsupervised tensor clustering framework on multiple-biomarker tensors to subdivide major lineages of MTBC into sublineages.

#### Subdivision of major lineages into sublineages

We subdivide each major lineage of MTBC into sublineages using multiple-biomarker tensors. For each major lineage, we generated the multiple-biomarker tensor using spoligotypes and MIRU types and applied multiway models to identify putative sublineages of each major lineage. Two multiway analysis methods were used: PARAFAC and Tucker3. Details of the methods and how the model parameters or components were selected can be found in the methods section. The validated multiway models with numbers of components for each major lineage are shown in Table 1. To evaluate the resulting clusters, we compared them to the published SpolDB4 families for each major lineage. The results are summarized in Table 2. We used the F-measure to measure how well the tensor sublineages match the SpolDB4 families with 1 indicating an exact match and 0 indicating no match. The average best-match stability is used to assess certainty of tensor sublineages respectively with 1 indicating highly stable clusters. For each major lineage, results show that the tensor analysis finds highly stable sublineages (the best-match stability is ≥84%) and that the number of sublineages found using tensors is close but not always identical to the number of SpolDB4 families.

**Table 1:** Number of components used in PARAFAC and Tucker3 models.

		PARAFAC		Tucker3	
Major Lineage	Tensor size				
		# Components	Core Consistency / Variance	# Components	Variance
*M. africanum*	64 × 22 × 12	3	95.08 / 93.33	[4 3 1]	91.94

*M. bovis*	102 × 34 × 12	2	100.00 / 86.02	[7 5 1]	91.05

East Asian (Beijing)	571 × 5 × 12	2	100.00 / 81.58	[3 4 2]	93.09

East-African Indian (CAS)	508 × 18 × 12	3	90.75 / 80.48	[6 6 4]	94.27

Indo-Oceanic	1023 × 28 × 12	5	92.99 / 80.35	[15 13 5]	95.55

Euro-American	4580 × 109 × 12	14	99.06 / 89.83	[14 13 5]	89.77

Number of components used in PARAFAC and Tucker3 models to fit the tensors for the datasets to be clustered. We used the core consistency diagnostic to validate PARAFAC models and percentage of explained variance to validate Tucker3 models.

**Table 2:** Number of SpolDB4 families and number of tensor sublineages for each major lineage

Major Lineage	# SpolDB4 families	# Tensor sublineages	F-measure	Best-match stability
*M. africanum*	4	4	0.66	1

*M. bovis*	5	3	0.71	1

East Asian (Beijing)	2	6	0.88	1

East-African Indian (CAS)	4	4	0.75	1

Indo-Oceanic	13	9	0.67	0.86

Euro-American	33	35	0.53	0.84

F-measure and average best-match stability are used to assess the agreement of the tensor sublineages to the SpolDB4 lineages and certainty of tensor sublineages respectively.

The F-measure values range from 53% to 88% indicating that the sublineages found by the tensors only partially overlap with those of SpolDB4. Recall that the SpolDB4 families were created by expert analysis using only spoligotypes and that analysis by alternative biomarkers such as SNP and LSP has led to alternative definitions of MTBC sublineages. The tensor sublineages are based on spoligotype and MIRU patterns, thus in some cases the tensor divides SpolDB4 families due to difference in MIRU patterns even if the spoligotypes match. In other cases, the tensor analysis merges the SpolDB4 families because the collective spoligotypes and MIRU patterns are very close. In some cases, the tensor analysis almost exactly reproduces a SpolDB4 family providing strong support for the existence of these families with no expert guidance. In addition, the MIRU patterns provide additional evidence for the existence of these distinct sublineages. Thus, multiway analysis of MTBC strains of each major lineage with multiple biomarkers leads to new sublineages and reaffirms existing ones. Further insight can be obtained by examining the putative sublineages for each major lineage, which is detailed next.

**Figure 3:**
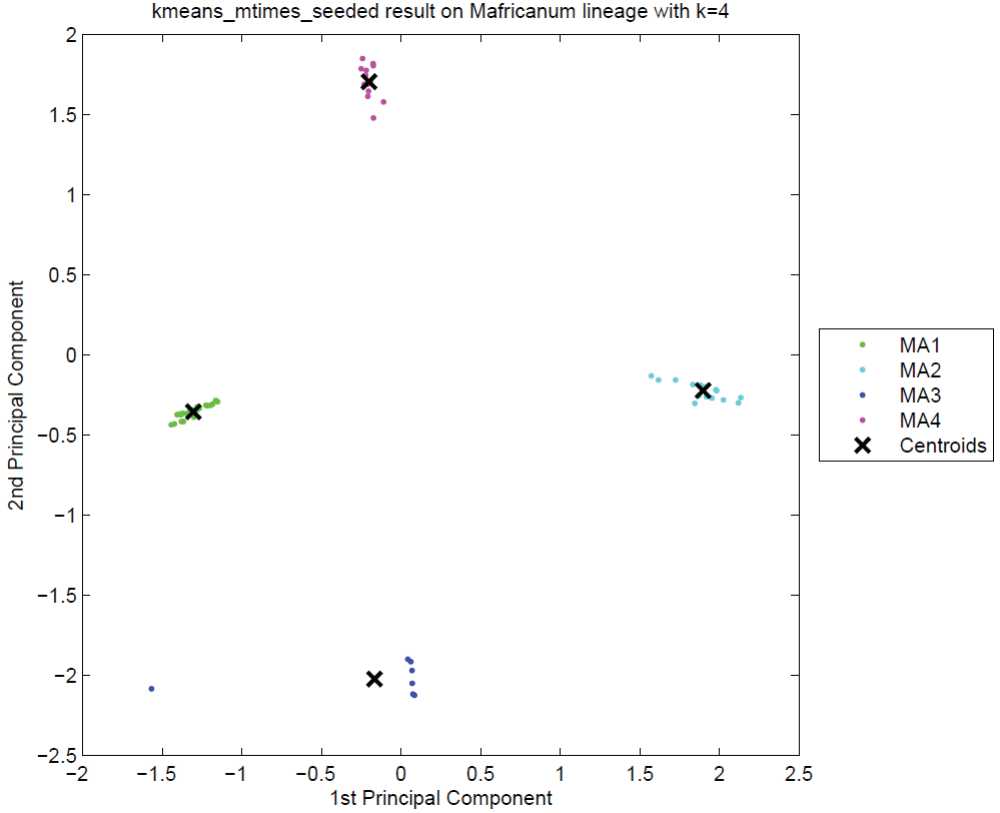
**The clustering plot of *M. africanum* strains** Clustering plot of *M. africanum* strains using Principal Component Analysis on the score matrix obtained from the PARAFAC model. Four putative tensor sublineages, MA1 to MA4, are clearly distinct along the principal component axes.

**Figure 4:**
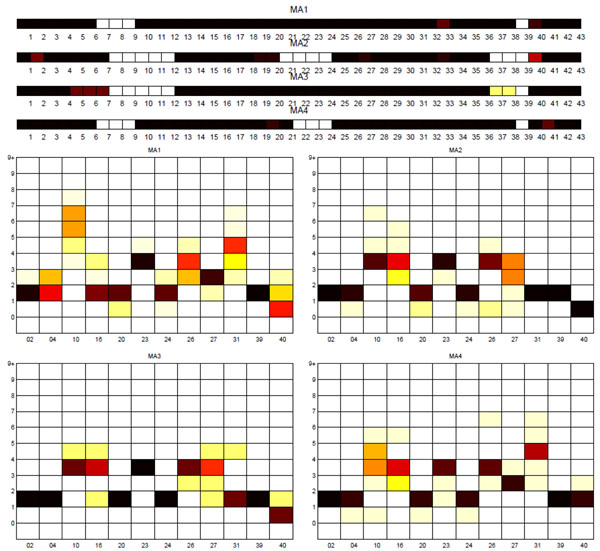
**Biomarker signatures of *M. africanum* tensor sublineages** Spoligotype and MIRU signatures of tensor sublineages of *M. africanum* strains. White indicates probability of 0 and black indicates probability of 1. Intermediate colors represent probabilities in the range (0,1). MA1 and MA4 are similar in their MIRU signatures, and MA4 strains lack spacers 22 through 24, in addition to the deletions of MA1 strains. MIRU signatures of MA2 and MA3 strains are also similar, and MA2 has an extra deletion, 21 through 24, in addition to the deletions of MA3 strains.

**Sublineage structure of *M. africanum*** The most stable clusters were produced using PARAFAC and it constructed four putative sublineages of *M. africanum*, denoted MA1 to MA4. Table 3 gives the stability of each sublineage and the correspondence between the tensor sublineages and the SpolDB4 families. These four putative sublineages are quite distinct as shown by the stability of 1 for each sublineage and the clear separation of the four sublineages in the PCA plot in Figure 3. Figure 4 shows heat maps representing the spoligotype and MIRU signatures for each tensor sublineage, with white indicating 0 probability and black indicating probability of 1.

**Table 3:** Confusion matrix of M. africanum strains

	MA1	MA2	MA3	MA4

Stability	1	1	1	1


AFRI	2	5	1	0

AFRI_1	21	0	0	16

AFRI_2	0	12	0	0

AFRI_3	0	1	6	0

Confusion matrix for 64 distinct *M. africanum* strains showing the correspondence between the SpolDB4 families and tensor sublineages. The stability of each tensor sublineage is given in the second row. All four *M. africanum* sublineages have a stability of 1, indicating that clear and distinct genetic diversity exists between the *M. africanum* sublineages. Each number in the table represents the number of strains that belong to associated SpolDB4 lineage in that row and associated tensor sublineage in that column.

The tensor sublineages strongly support the existence of the SpolDB4 AFRI_1, AFRI_2 and AFRI_3 families and show that the AFRI family is composed of these three families. With an F-measure of 66%, the tensor sublineages differ markedly from the SpolDB4 families for the *M. africanum* lineage. The AFRI family results largely explain this difference – AFRI is spread across three tensor sublineages. Disregarding AFRI, sublineages MA2 and MA3 match families AFRI_2 and AFRI_3 respectively. Interestingly, AFRI_1 is further subdivided into sublineages MA1 and MA4. The spoligotypes in MA1 and MA4 differ by only one contiguous deletion of spacers 22 through 24, but their MIRU signatures clearly distinguish them especially in MIRU loci 10, 16 and 40. The tensor indicates that the AFRI sublineage classification defines somewhat generic *M. africanum* strains that can be distinctly placed in the groups MA1 (part of AFRI_1), MA4 (other part of AFRI_1), MA2 (AFRI_2) and MA3 (AFRI_3).

The MIRU-VNTR*plus* labels, determined on the basis of LSPs, indicate that there are two sublineages, West African 1 and West African 2, within *M. africanum.* Table 4 indicates the correspondence between the tensor sublineages and MIRU-VNTR*plus* labels. MA1 and MA4 correspond to West African 2 and MA2 corresponds to West African 1. There is no data labeled by MIRU-VNTR*plus* in MA3, but we speculate that it is West African 1 since MA2 and MA3 have more closely related MIRU and spoligotype signatures.

**Table 4:** Confusion matrix of distinct *M. africanum* strains based on MIRUVNTRplus sublineages

	MA1	MA2	MA3	MA4
West African 1	0	5	0	0

West African 2	21	0	0	16

Unspecified	2	13	7	0

Confusion matrix for 64 distinct *M. africanum* strains showing the correspondence between the West African 1 and 2 sublineages and tensor sublineages. For the data not from MIRU-VNTR*plus*, the lineage is indicated as unspecified.

**Figure 5:**
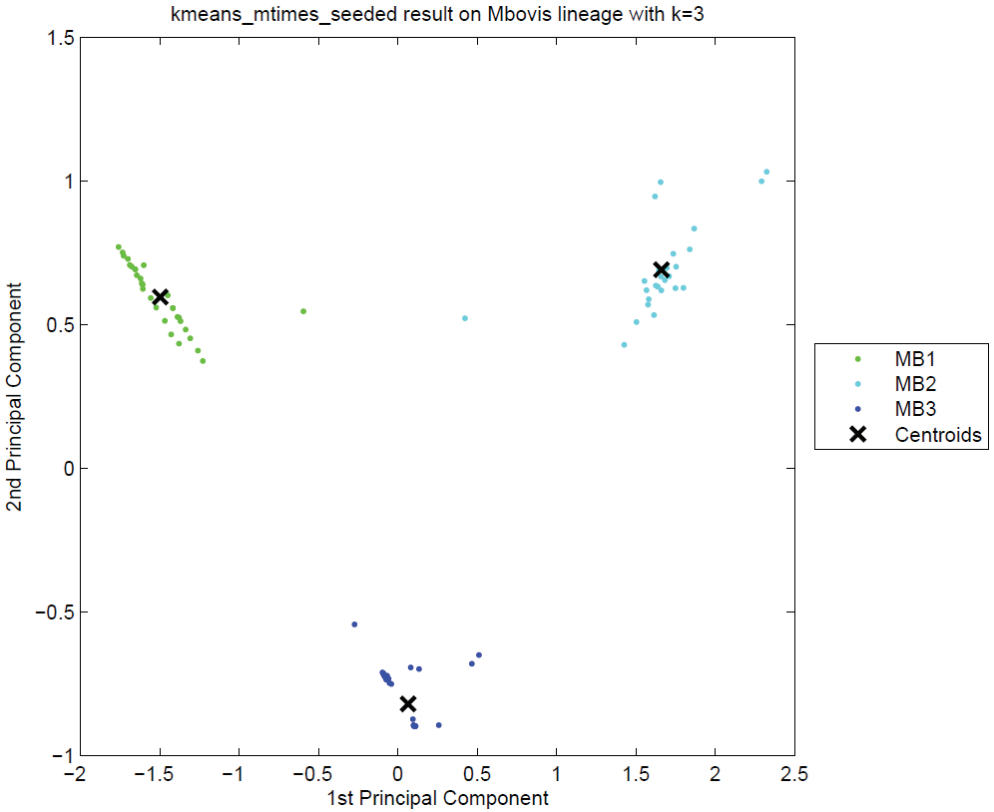
**The clustering plot of *M. bovis* strains** Clustering plot of *M. bovis* strains using Principal Component Analysis. Three putative tensor sublineages, MB1 to MB3, are clearly separated.

**Figure 6:**
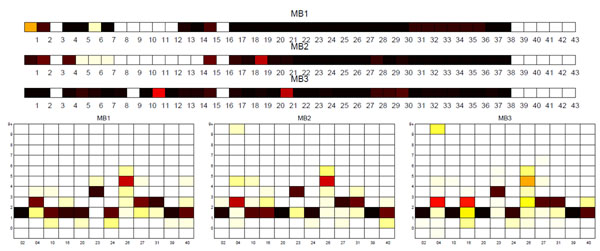
**Biomarker signatures of *M. bovis* tensor sublineages** Spoligotype and MIRU signatures of tensor sublineages of *M. bovis* strains. Although MIRU signatures of MB1 and MB2 strains are similar, spoligotype signatures of MB1 and MB2 strains are clearly distinguishable by extra deletions of 13 through 14 in all MB2 strains, and deletions of 5 through 7 in some MB2 strains.

**Sublineage structure of *M. bovis*** PARAFAC generated the most stable clusters and constructed 3 sublin-eages for *M. bovis*, MB1, MB2, and MB3, while the dataset contains 5 SpolDB4 families, BOV, BOVIS1, BOVIS1_BCG, BOVIS2, and BOVIS3. Table 5 gives the correspondence between the tensor sublineages and the SpolDB4 families. All clusters have perfect stability and are well distinguished in the PCA plot in Figure 5. Figure 6 shows heat maps representing the spoligotype and MIRU type signatures of tensor sublineages. Much like the *M. africanum* SpolDB4 AFRI family, the BOV family defines a generic *M. bovis* sublineage that spreads across all three tensor sublineages. Disregarding BOV, MB3 consists of all of BOVIS1 and BOVIS1_BCG strains. Since BOVIS1_BCG is the attenuated bacillus Calmette-Guérin (BCG) vaccine strain, it is difficult to distinguish it from BOVIS1 using only MIRU patterns and spoligotypes. Therefore, the merger of BOVIS1 and BOVIS1 BCG is expected given the genetic similarity between the two groups of strains. Disregarding BOV, the MB1 and MB2 sublineages exactly match the SpolDB4 families BOVIS2 and BOVIS3 respectively.

**Table 5:** Confusion matrix of M. bovis strains

	MB1	MB2	MB3

Stability	1	1	1


BOV	7	5	5

BOVIS1	0	0	29

BOVIS1_BCG	0	0	11

BOVIS2	24	0	0

BOVIS3	0	21	0

Confusion matrix of *M. bovis* strains clustered into 3 groups using PARAFAC. Correct labels are SpolDB4 labels on the rows, and tensor sublineages are represented by each column. Stability of 1 for the tensor sublineages indicates that they have clear and marked differences based on their genotype. MB1 contains all BOVIS2 strains, MB2 contains all BOVIS3 strains, and MB3 contains all BOVIS1 and BOVIS1_BCG strains.

**Figure 7:**
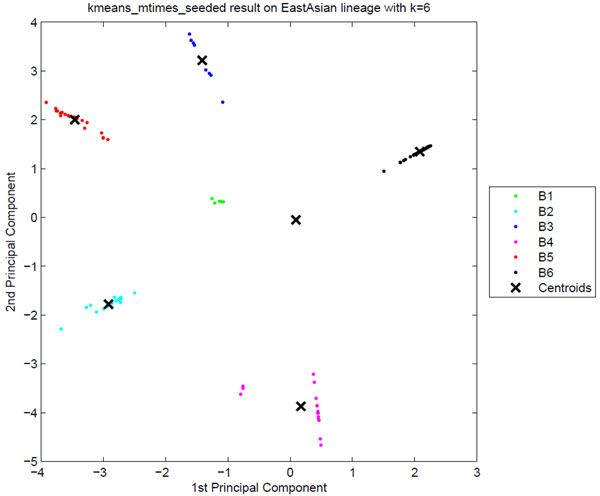
**The clustering plot of East Asian (Beijing) strains** Clustering plot of East Asian (Beijing) strains using Principal Component Analysis. Six putative tensor sublineages, B1 to B6, are clearly distinct.

**Figure 8:**
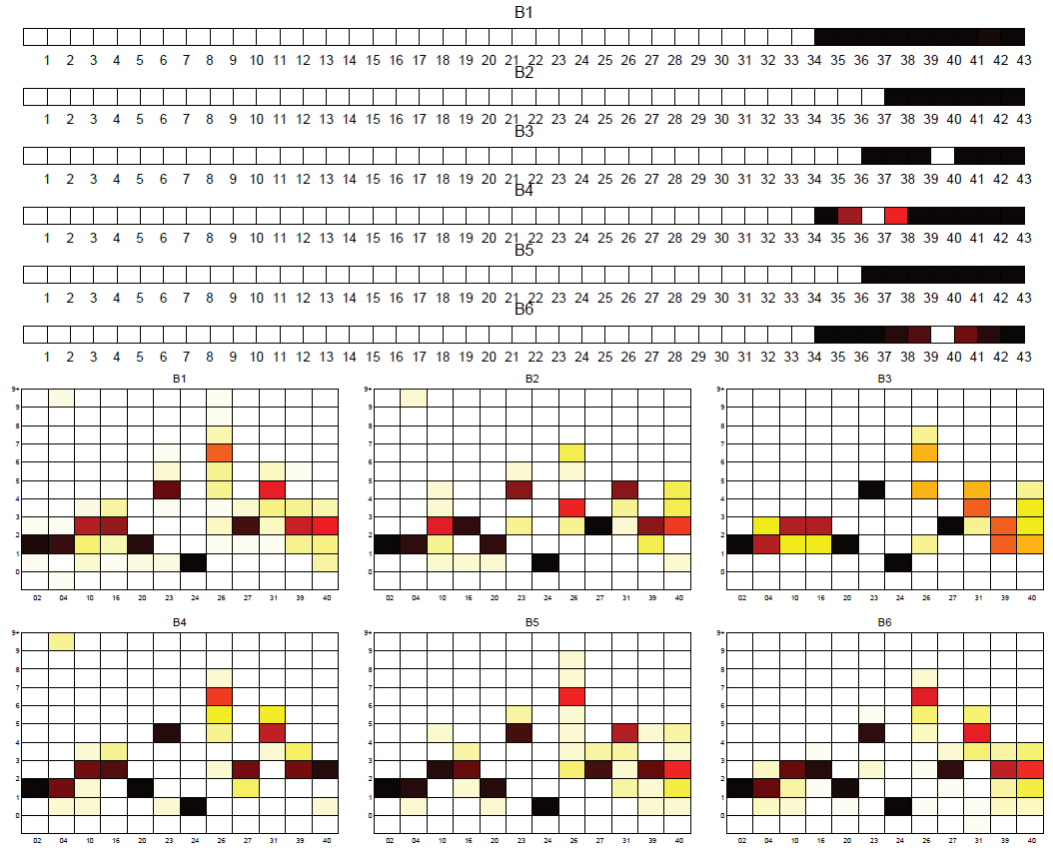
**Biomarker signatures of East Asian (Beijing) tensor sublineages** Spoligotype and MIRU signatures of tensor sublineages of East Asian (Beijing) strains. Tensor sublineages B1, B4, B6 include BEIJING strains and sublineages B2, B3, B5 include BEIJING-LIKE strains.

**Sublineage structure of East Asian (Beijing)** The most stable clusters are produced by Tucker3 and it constructs six distinct sublineages of East Asian (Beijing), denoted B1 through B6. The variability in the spoligotypes of East Asian is limited to spacers 35 through 43 since all East Asian strains have spacers 1 to 34 absent. Since the SpolDB4 classification is based only on spoligotypes, the limited variability allows only two families, BEIJING and BEIJING-LIKE. Table 6 shows the correspondence between tensor sublin-eages and the SpolDB4 families. The clustering plot of tensor sublineages is shown in Figure 7. Heat maps representing the spoligotype and MIRU type signatures of tensor sublineages are shown in Figure 8. The tensor cleanly subdivides BEIJING into three sublineages B1, B4 and B6, all with stability 1. Spoligotype signatures of these sublineages differ. B1 strains have spacers 35 through 43 present, whereas B4 strains lack spacer 37, and B6 strains lack spacer 40. MIRU signature of sublineage B4 is clearly distinct in MIRU locus 40, having 3 repeats for most strains. The tensor subdivides the BEIJING-LIKE into sublineages B2, B3 and B5, each with distinct spoligotype signature. They all lack spacers 35 through 36. In addition, B2 strains lack spacer 37, and B3 strains lack spacer 40. Thus, the tensor strongly supports the existence of BEIJING and BEIJING-LIKE families, but also suggests that they can be further subdivided.

**Table 6:** Confusion matrix of East Asian (Beijing) strains

	B1	B2	B3	B4	B5	B6

Stability	1	1	1	1	1	1


BEIJING	468	0	0	18	0	41

BEIJING-LIKE	0	16	8	0	20	0

Confusion matrix of East Asian (Beijing) strains clustered into 6 groups using Tucker3. Correct labels are SpolDB4 labels on the rows, and tensor sublineages are represented by each column. The six highly stable tensor sublineages are indicative of additional genetic diversity within the BEIJING and BEIJING-LIKE sublineages.

**Figure 9:**
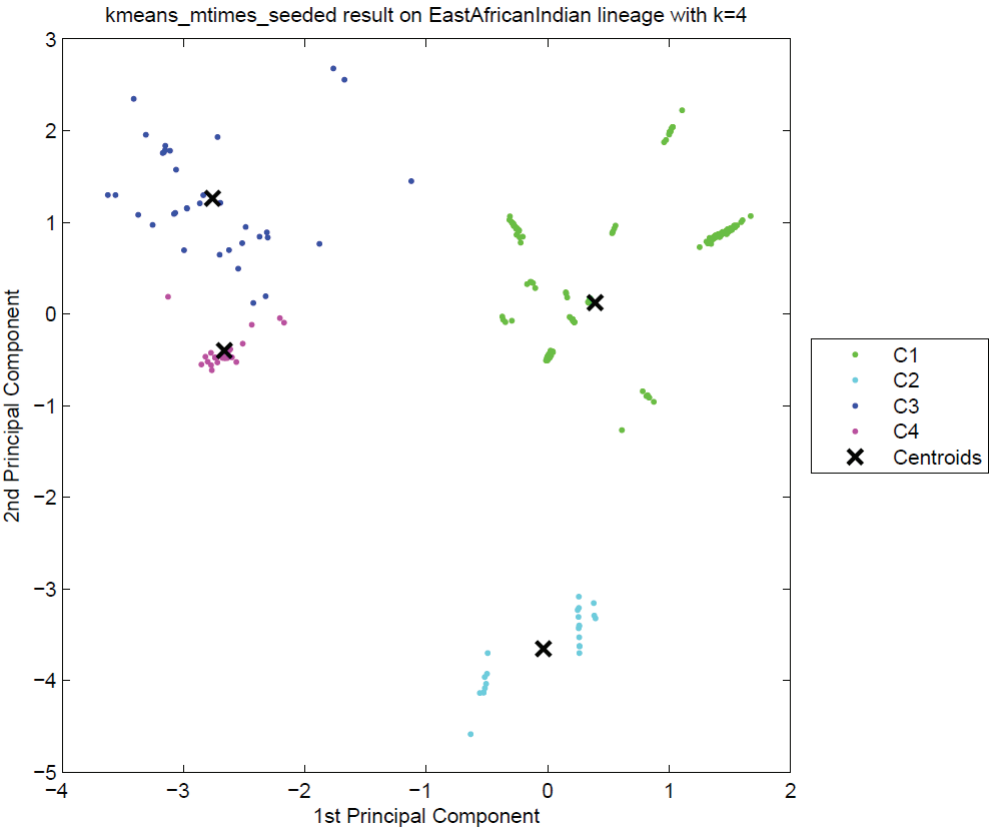
**The clustering plot of East-African Indian (CAS) strains** Clustering plot of East-African Indian (CAS) strains using Principal Component Analysis. Four putative tensor sublineages, C1 to C4, are clearly distinct.

**Figure 10:**
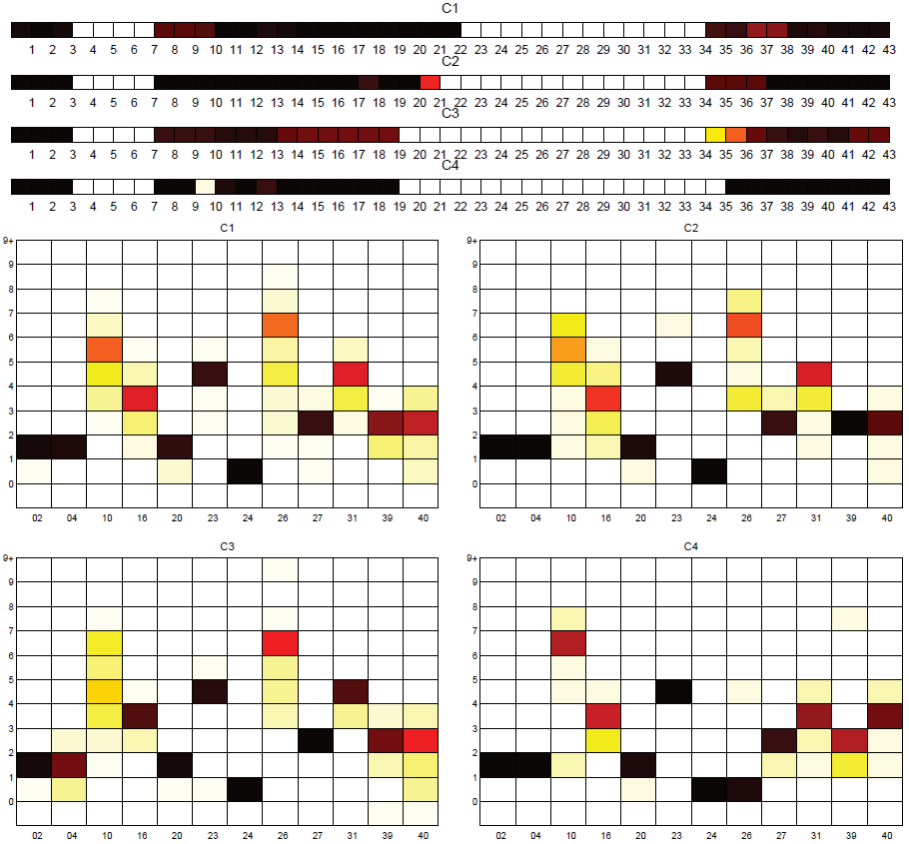
**Biomarker signatures of East-African Indian (CAS) tensor sublineages** Spoligotype and MIRU signatures of tensor sublineages of East-African Indian (CAS) strains. In addition to deletions in C1 strains, C2 strains lack spacer 22. In addition to deletions in C3 strains, C4 strains lack spacer 35 and have only 1 repeat in MIRU 26. C2 and C3 strains are very close in their MIRU signature, but they differ by variations in MIRU locus 10.

**Sublineage structure of East-African Indian (CAS)** Tucker3 generated the most stable clusters and it constructed four distinct sublineages for East-African Indian (also known as CAS) denoted C1, C2, C3, and C4. The strains are also labeled with four SpolDB4 lineages: CAS, CAS1_DELHI, CAS1_KILI and CAS2. Table 7 shows the correspondence of tensor sublineages and SpolDB4 families. Figure 9 shows the clustering plot of tensor sublineages and Figure 10 shows spoligotype and MIRU type signatures of tensor sublineages. All sublineages are highly stable with stability 1. Much like with AFRI and BOV, the generic CAS family is divided across all tensor sublineages. C3 only contains CAS strains. Disregarding CAS, C1 contains most CAS1 DELHI strains and all CAS2 strains. C4 contains all CAS1_KILI strains. C2 contains 2 CAS1_DELHI strains, but the vast majority (331 strains) of CAS1_DELHI strains fall in C1. In addition to the common deletions of East-African Indian (CAS) strains, C2 strains lack spacer 22, C3 strains lack spacers 20 through 22, and C4 strains lack spacers 20 through 22 and spacer 35. Variabilities in MIRU loci 10, 26, 31 and 40 are also key to defining differences in the sublineages. C2 and C3 strains differ by variations in MIRU locus 10. C4 strains which include all CAS1_KILI strains exhibit a very distinct MIRU signature compared to other tensor sublineages, especially in MIRU locus 26.

**Table 7:** Confusion matrix of East-African Indian (CAS) strains

	C1	C2	C3	C4

Stability	1	1	1	1


CAS	50	21	35	1

CAS1_DELHI	331	2	0	0

CAS1_KILI	0	0	0	23

CAS2	45	0	0	0

Confusion matrix of East-African Indian (CAS) strains clustered into 4 groups using Tucker3. Correct labels are SpolDB4 labels on the rows, and tensor sublineages are represented by each column.

**Figure 11:**
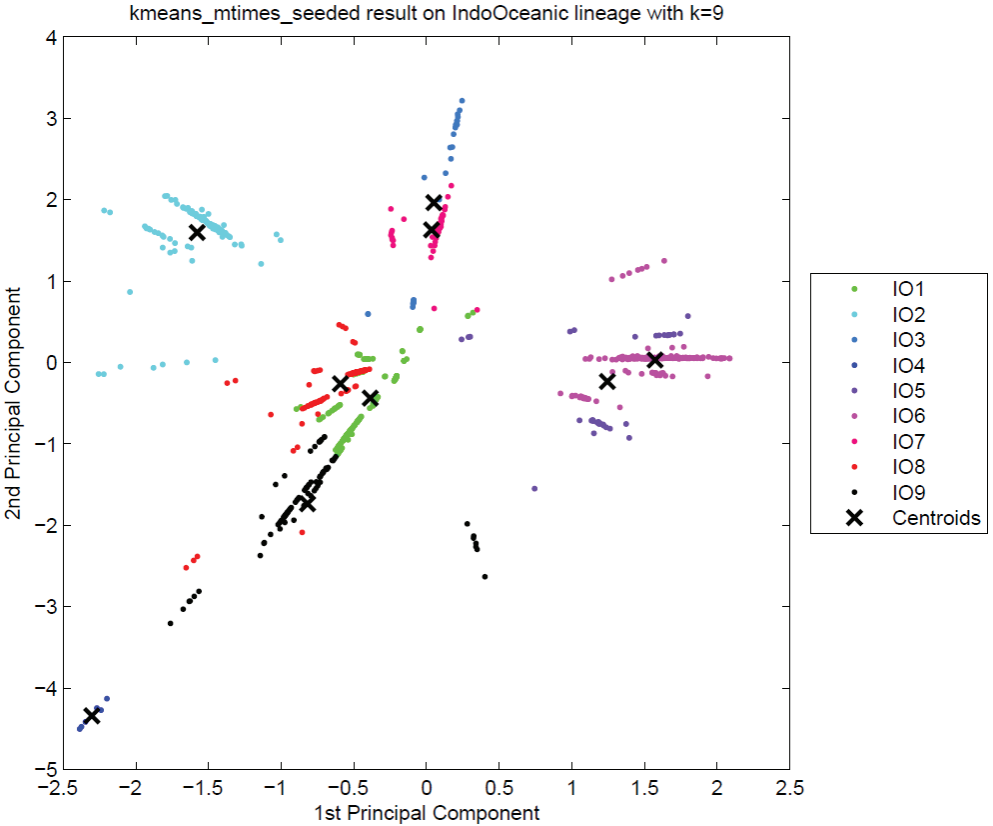
**The clustering plot of Indo-Oceanic strains** Clustering plot of Indo-Oceanic strains labeled by putative tensor sublineages using Principal Component Analysis. The tensor sublineages are not as distinct as they were for the previously analyzed major lineages, implying that the tensor sublineages are well distinguished in the PCA plot if they are stable.

**Figure 12:**
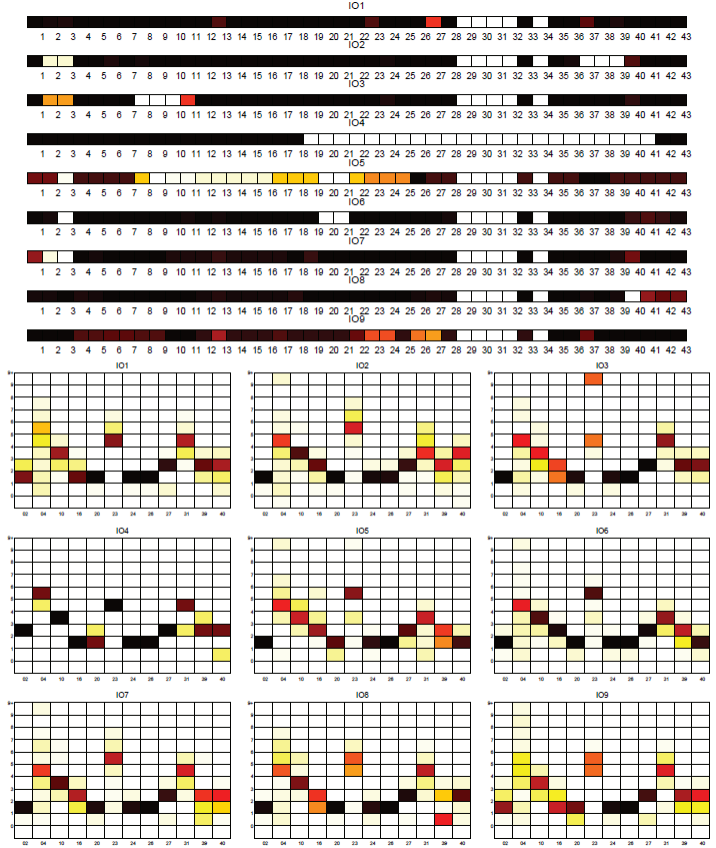
**Biomarker signatures of Indo-Oceanic tensor sublineages** Spoligotype and MIRU signatures of tensor sublineages of Indo-Oceanic strains.

**Sublineage structure of Indo-Oceanic** PARAFAC found the most stable clusters and it constructs nine distinct putative sublineages for Indo-Oceanic, denoted IO1 to IO9, while the dataset has thirteen SpolDB4 lineages. Table 8 shows the correspondence of tensor sublineages and SpolDB4 families. Figure 11 shows the clustering plot of tensor sublineages and Figure 12 shows spoligotype and MIRU signatures of tensor sublineages. The EAI5 family acts much like the CAS, BOV, and AFRI families, spreading across all the Indo-Oceanic sublineages except IO4. The small MANU1 family also spreads across four sublineages. The existence of the MANU1 family has not been well established by other biomarkers. Disregarding these two troubling families, the tensor sublineages correspond closely to the SpolDB4 families. Table 8 shows that there is almost a one-to-one mapping between most SpolDB4 lineages and Indo-Oceanic tensor sublineages. Specifically, the mapping between the most stable clusters (with sublineage stability) and the families are: IO1 (.94) equals EAI6_BDG1, IO2 (1) equals EAI3_IND, IO4 (1) equals ZERO, and IO6 (.91) equals most of EAI2_MANILLA. All EAI strains are in IO9 (.77), all EAI1 strains are in IO8 (.86), all MICROTI strains are in IO5 (0.56), and all ZERO strains are in IO4. All EAI2_NTB strains are in IO5, all EAI3_IND strains are in IO2, and all EAI8_MDG strains are in IO7 (.84). EAI2_MANILLA is divided into two sublineages: 11 strains in IO5, 265 strains in IO6. While the spoligotype and MIRU signatures show that there are distinct EAI5 subgroups, the definition of the EAI5 and MANU1 groups are not well supported by the tensor analysis. They may represent a more generic sublineage that is further subdivided. Distinct patterns are observable in the spoligotype and MIRU signatures for most of the tensor sublineages.

**Table 8:** Confusion matrix of Indo-Oceanic strains

	IO1	IO2	IO3	IO4	IO5	IO6	IO7	IO8	IO9

Stability	0.94	1	0.90	1	0.56	0.91	0.84	0.86	0.77


EAI	0	0	0	0	0	0	0	0	6

EAI1	0	0	0	0	0	0	0	2	0

EAI1_SOM	0	0	2	0	0	0	8	107	0

EAI2_MANILLA	0	0	0	0	11	265	0	0	0

EAI2_NTB	0	0	0	0	15	0	0	0	0

EAI3_IND	0	105	0	0	0	0	0	0	0

EAI4_VNM	0	0	0	0	0	0	0	3	42

EAI5	231	24	26	0	3	10	35	32	31

EAI6_BGD1	33	0	0	0	0	0	0	0	10

EAI8_MDG	0	0	0	0	0	0	4	0	0

MANU1	1	0	0	0	0	5	0	2	1

MICROTI	0	0	0	0	3	0	0	0	0

ZERO	0	0	0	6	0	0	0	0	0

Confusion matrix of Indo-Oceanic strains clustered into 9 groups using PARAFAC. Correct labels are SpolDB4 labels on the rows, and tensor sublineages are represented by each column. SpolDB4 lineages except EAI5 and MANU1 map to distinct tensor sublineages.

**Figure 13:**
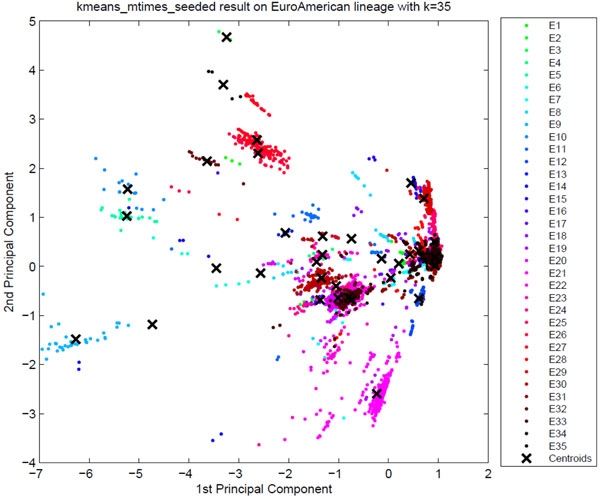
**The clustering plot of Euro-American strains** Clustering plot of Euro-American strains labeled by 35 tensor sublineages using Principal Component Analysis. The tensor sublineages are not as distinct as they were for the previously analyzed major lineages, reflecting the variability in the tensor cluster stability. It may also be due to the anticipated hierarchical structure in Euro-American strains.

**Figure 14:**
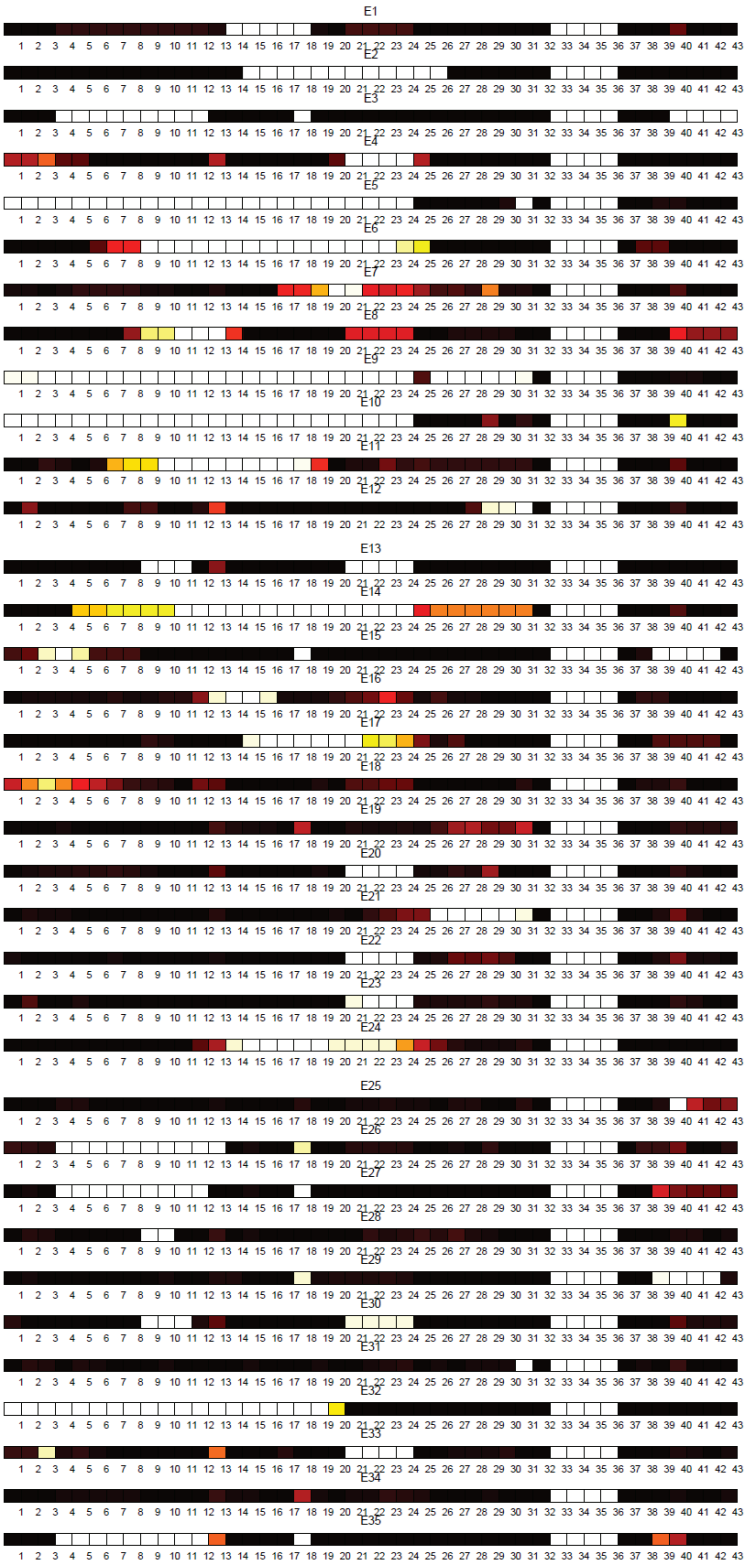
**Spoligotype signatures of Euro-American tensor sublineages** Spoligotype signatures of tensor sublineages of Euro-American strains.

**Figure 15:**
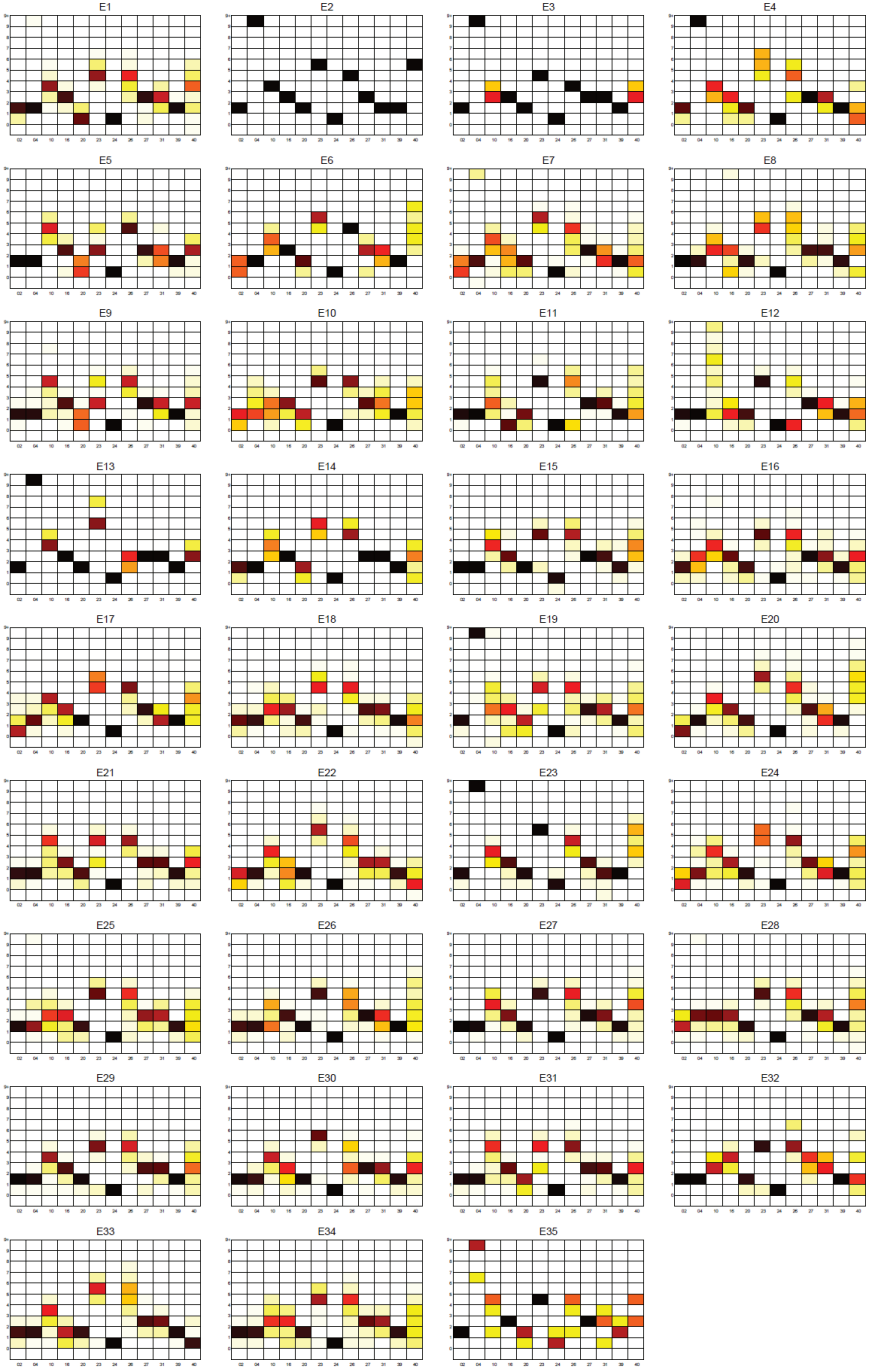
**MIRU signatures of Euro-American tensor sublineages** MIRU signatures of tensor sublineages of Euro-American strains.

**Sublineage structure of Euro-American** Tucker3 found the most stable clusters and it generates 35 sublineages for Euro-American, denoted E1 to E35, while there are 33 SpolDB4 lineages labeled Euro-American. See additional file 1 for the confusion matrix of Euro-American strains that shows the correspondence of tensor sublineages and SpolDB4 families. Figure 13 shows the clustering plot of tensor sublineages. Figure 14 and Figure 15 show the spoligotype and MIRU signatures of tensor sublineages respectively.

Strains belonging to families H2, H37Rv, LAM12_MAD1, T1 (Tuscany variant), T1_RUS2, T4, T5_MAD2, and T5_RUS1 are clustered in tensor sublineages E9, E7, E8, E24, E11, E34, E34, and E17 respectively. In contrast, the T1 family, an ancestor strain family, is distributed across 25 tensor sublin-eages, with most T1 strains in E34. Sublineage stability is above .90 for 18 tensor sublineages. Spoligotype and MIRU signatures of sublineages suggest either subdivision or merging of SpolDB4 families. For instance, tensor sublineages E2, E6, and E32 include T1 strains only. In addition to common spacer deletions of Euro-American strains, E2 strains lack spacers 15 through 26, E6 strains lack spacers 9 through 23, and E32 strains lack spacers 1 through 19, which are all variations in spoligotype signatures of T1 strains. This sublineage classification further subdivides the poorly-defined ancestor T1 family. Strains of LAM families on the other hand are grouped in 17 tensor sublineages. Prior studies have found that LAM Rio strains identified by SNPs are found in multiple SpolDB4 lineages [20]. Therefore, it is expected that the use of multiple biomarkers leads to subdivision or merging of some SpolDB4 families.

Although most stable clusters of the Euro-American strain dataset are found using best-match stability, the DD-weighted gap statistic plot has multiple peaks. DD-weighted gap statistic, detailed in the methods section, is a cluster validity measure which is also used for detecting hierarchical structure in the datasets. Multiple peaks in DD-weighted gap statistic plot suggest that the Euro-American dataset may have a multilevel hierarchical structure. Model order selection with randomized maps by Bertoni and Valentini can be used to detect the hierarchical structure in the Euro-American dataset [21].

We used the unsupervised tensor clustering framework to cluster MTBC strains of major lineages into sublineages. Next, we turn our attention to supervised tensor learning methods on multiple-biomarker tensors to classify strains into major lineages.

#### Classification of MTBC strains into major lineages using two-way and multiway supervised learning

Multiple-biomarker tensors can be used in supervised classification models as well as in unsupervised models. We use multiway partial least squares (N-PLS) on multiple-biomarker tensors to predict major MTBC lineages [22]. In our experiments, we used spoligotype and MIRU as biomarkers and predicted the six major lineages using the same data as for the above unsupervised learning experiments combined into a single dataset. More specifically, we used 12 spoligotype deletions found informative in major lineage classification combined with 12-loci MIRU [23]. We predicted major lineages with the N-PLS multiway method and compared it with standard two-way PLS and prior results for conformal Bayesian Networks [4]. Table 9 shows the average testing F-measure as estimated by 5-fold cross-validation. We generate the multiple-biomarker tensor using 12 spoligotype deletions and 12-loci MIRU with one additional bit indicating whether the at least one MIRU pattern includes letter rather than number of repeats, and create a predictive model using the N-PLS multiway method. The model for standard 2-way PLS is created by representing the data as a matrix with columns corresponding to 12 spoligotype deletions and 12-loci MIRU with the additional indicator bit, and rows corresponding to MTBC strains. The number of latent variables for both N-PLS and PLS are selected by inner 4-fold cross-validation of the training set data only.

**Table 9:** Multiway N-PLS and standard two-way PLS classification accuracy results

Method	Average F-measure
N-PLS	0.9961 ± 0.0009

Standard PLS	0.9955 ± 0.0017

Conformal Bayes Net	0.9897

Multiway N-PLS and standard two-way PLS classification accuracy results when 12 spoligotype deletions and MIRU patterns are used to classify MTBC strains into major lineages. The excellent results compare favorably to prior results based on a conformal Bayesian Network in [4].

We compare N-PLS, standard PLS and Conformal Bayes Network (CBN) methods by F-measure of major lineage classification and see that they are accurate predictive models with no significant difference between the approaches. Table 9 shows the F-measure values for N-PLS, standard PLS and CBN. The average F-measure of major lineage prediction on the same data using the CBN is 0.9897 [4]. This shows that N-PLS and standard PLS methods predict major lineages as accurately as CBN, with a slightly better average F-measure value. All three methods achieve outstanding results for major lineage classification with no significant difference between approaches.

## Conclusions

This study investigates multiple-biomarker tensors and illustrates how they can be used for both unsupervised and supervised learning models. First, a novel clustering framework is used to analyze the sublineage structure of MTBC strains based on multiple biomarkers. We generated multiple-biomarker tensors to represent multiple biomarkers of the MTBC genome and used multiway models for dimensionality reduction. The multiway representation determines a transformation of the data that captures similarities and differences between strains based on two distinct biomarkers. We clustered MTBC strains based on the transformed data using improved k-means clustering and validated clustering results. We evaluated the sublineage structure of major lineages of MTBC and found similarities and clear distinctions in our subdivision of major lineages compared to the SpolDB4 classification. Simultaneous analysis of spoligotype and MIRU through multiple-biomarker tensors and clustering of MTBC strains leads to coherent sublineages within major lineages with clear and distinctive spoligotype and MIRU signatures. Second, we demonstrated how the multiple-biomarker tensor can be used to predict major lineages with extremely high accuracy competitive with other approaches. We show that 3-way PLS, 2-way PLS and CBN models are accurate major lineage predictors for MTBC strains.

The tensor clustering framework is flexible and can be applied to any multidimensional strain data. The design of the resulting tensor depends on the question to be answered. In this study, multiple-biomarker tensors are designed to find groups of MTBC strains. Thus, the application of the tensor clustering framework on multiple-biomarker tensors leads to sublineages of MTBC within major lineages. The multiple-biomarker tensor is further validated by the fact that it can used to predict known major lineages with high accuracy using N-PLS. N-PLS with multiple-biomarker tensors can be used for semi-supervised learning as well. This can be useful for learning predictive models for sublineages in which only part of the data is labeled with sublineages and the other part of the data has no labels. This may result in more reliable and accurate classifiers of MTBC sublineages, and the resulting sublineage classifiers would be a significant enhancement to TB control, epidemiology and research. We leave this to future work.

The tensor clustering framework used in this study can be further extended to find subgroups of MTBC strains based on other biomarkers such as RFLP and SNPs. 15-loci MIRU and 24-loci MIRU patterns can also be used to represent MTBC genomes with multiple-biomarker tensors. Moreover, more than two biomarkers can be used in the MTBC genome representation. But, ambiguity in the tensor entries is an open question that needs to be solved in the tensor representation when more than two biomarkers are used. Addition of new biomarkers will increase the number of modes of the multiple-biomarker tensor, but the multiway analysis methods will remain the same.

Other questions of interest can be addressed by designing and analyzing host-pathogen tensors to examine the relationship of the pathogen genotype with host (or equivalent) attributes to examine questions of interest. For example, since the MTBC sublineages are known to be highly geographically dependent, a tensor which combines the pathogen genotype with the country of birth of the host may reveal additional sublineage structure and transmission patterns. A tensor combining MTBC genotype and host disease phenotype such as site of infection and drug resistance could be used to analyze MTBC genotype/phenotype relations.

## Methods

### Tensor Clustering Framework (TCF)

**Figure 16:**
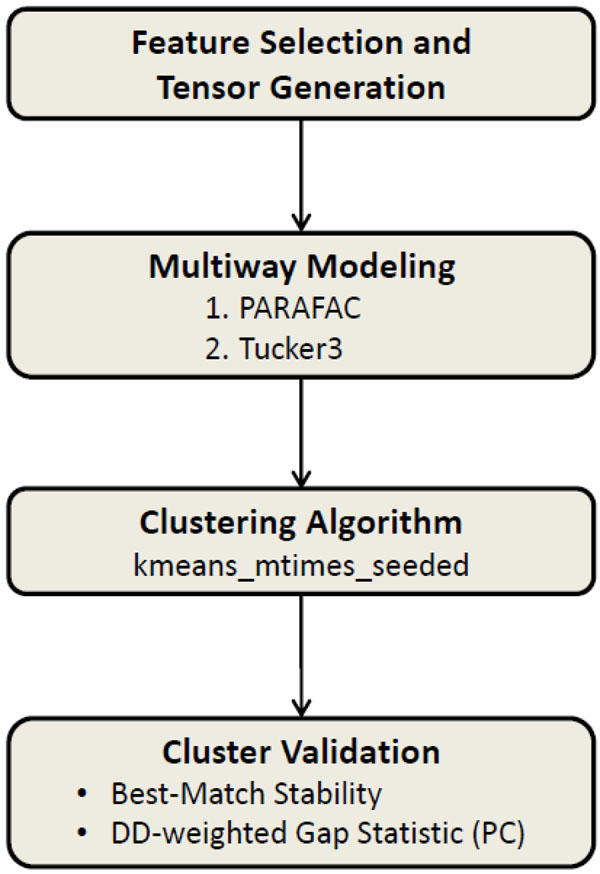
**Tensor clustering framework** Clustering framework of MTBC strains. High-dimensional genotype data are decomposed into two-dimensional arrays using multiway models, which are then used as input to the kmeans_mtimes_seeded algorithm. Clusterings are validated using best-match stability. In case of a tie, the DD-weighted gap statistic is used to pick the number of clusters.

Clustering MTBC strains based on multiple-biomarker tensors consists of a sequence of steps. First, we find informative feature set of spoligotype deletions and generate a tensor. Second, we apply multiway models on the tensor and get a score matrix for the strain mode. Third, we use this score matrix to determine the similarity between strains, and cluster them using a stable version of k-means. In the final step, we evaluate the clustering results using cluster validity indices. This stepwise clustering framework is outlined in Figure 16. We describe the steps of the tensor clustering framework in this section.

#### Datasets

The dataset comprises 6848 distinct MTBC strains as determined by spoligotype and 12-loci MIRU, labeled with major lineages and SpolDB4 families. The strains are mainly from the CDC dataset - a database collected by the CDC from 2004-2008 labeled with the major lineages collected by the TB-Insight project (http://tbinsight.cs.rpi.edu/) that was previously studied in [4]. We also used the MIRU-VNTR*plus* dataset from www.MIRUVNTRplus.org which is labeled with SpolDB4 lineages and sublineages. The original SpolDB4 labeled dataset provided in an online supplement [6] contains only spoligotypes. We found all occurrences of these spoligotypes in the CDC and MIRU-VNTR*plus* dataset and constructed a database with spoligotype and MIRU patterns, with major lineages as determined by CDC, and sublineages as given in the SpolDB4 database [6]. The numbers of strains for each major lineage in the resulting dataset are shown in Table 10. We created 6 datasets from the CDC+MIRU-VNTR*plus* dataset, one for each major lineage. These same 6 major lineage datasets were merged into one for the supervised learning experiment.

**Table 10:** Data statistics by major lineage

Major lineage	# Strains	# Spoligotype deletions
*M. africanum*	64	22

*M. bovis*	102	34

East Asian (Beijing)	571	5

East-African Indian(CAS)	508	18

Indo-Oceanic	1023	28

Euro-American	4580	109

Numbers of strains in each major lineage of CDC+MIRU-VNTR*plus* dataset and numbers of spoligotype deletions identified by the feature selection algorithm.

#### Feature Selection and Tensor Generation

**Feature Selection** The spoligotype pattern captures the variability in the DR locus of the MTBC genome. A spoligotype consists of 43 spacers represented as a 43-bit binary sequence, and according to the hidden parent assumption, one or more contiguous spacers can be lost in a deletion event, but rarely gained [8,24]. Therefore, there are  possible deletions of lengths varying from 1 to 43 in a spoligotype. Only subsets of spoligotypedeletions are required for effective discrimination of MTBC strains. A set of 12 deletion sequences of spoligotypes reported by Shabbeer et al. have proven to be good discriminator spacer deletions for major lineage classification [23]. These 12 deletion sequences are used in the supervised learning study. Another set of 81 deletion sequences of spoligotypes reported by Brudey et al. have proven to be good discriminator spacer deletions for SpolDB4 sublineage classification [6].

Within the TCF, we built a feature selection algorithm to find spacer deletions that are informative. This insures that the results are not biased by a priori selection of spoligotype deletions. Given a set of spoligotypes, we first calculate the frequency *f_i_*, *i* = 1,‥, 946, of each possible deletion among the spoligotypes of strains. If *f_i_* = 1, the deletion is a common deletion. If 0 ≤ *f_i_* <*threshold*, the deletion is a nonexistent deletion, where *threshold* is data dependent and *threshold* = 0.05 is used by default. The deletions with frequency *f_i_* such that threshold ≤ *f_i_* < 1 are uncommon deletions. In the second step, we iterate through the set of uncommon deletions *U*, and remove an uncommon deletion *u* ∈ *U*, if there exists a common deletion *c* ∈ *C* which is a substring of u. We assign the final set of uncommon deletions as the feature set. Using the final feature set, we determine spoligotype deletions that are effective in discriminating the strains of the dataset. Algorithm 1 summarizes the feature selection procedure. Numbers of spoligotype deletions for each major lineage, found informative by the feature selection algorithm, are given in Table 10.

**Tensor Generation** We generated multiple-biomarker tensors using two biomarkers, spoligotype deletions and MIRU patterns, as explained earlier. The spoligotype deletions found informative by the feature selection algorithm are used in the generation of multiple-biomarker tensors. The multiple-biomarker tensor is of the form *Strains* × *Spoligotype deletions* × *MIRU patterns*. We used the tensor clustering framework on multiple-biomarker tensors to cluster strains.

#### Multiway modeling

Multiway models are needed to fit a model to multiway arrays. We used PARAFAC and Tucker3 techniques to model the tensors. We determined the number of components for each model to ensure a bound on the explained variance of data.

**Multiway models** We used PARAFAC and Tucker3 models to explain the tensor with high accuracy. Multiway modeling of tensors was carried out using the *n-wayToolbox* of MATLAB by Bro et al. and the *PLS toolbox*[25,26].

#### PARAFAC

**Figure 17:**
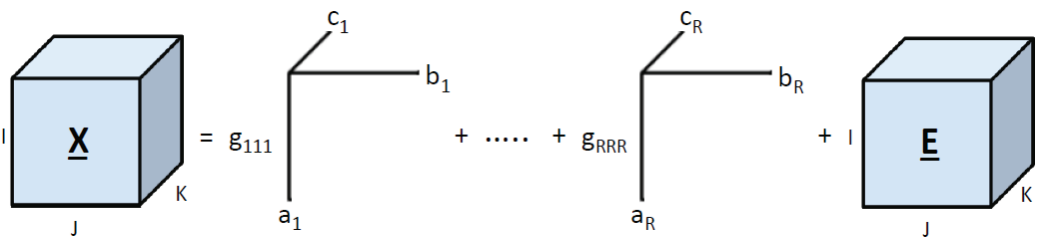
**PARAFAC model** PARAFAC model of a three-way array **X** with *R* components. The tensor is modeled as a linear combination of rank-one tensors for each mode.

PARAFAC is a generalization of singular value decomposition to multiway data [27,28]. A 3-way array **X** ∈ ℝ*^I^*^×^*^J^*^×^*^K^* is modeled by an *R*-component PARAFAC model as follows:

where **A** ∈ ℝ*^I^*^×^*^R^*, **B** ∈ ℝ*^J^*^×^*^R^*, **C** ∈ ℝ*^K^*^×^*^R^* are component matrices of first, second, and third mode. **G** ∈ ℝ*^R^*^×^*^R^*^×^*^R^* is the core array, and **E** ∈ ℝ*^I^*^×^*^J^*^×^*^K^* is the residual term containing all unexplained variation. A description of the PARAFAC model is shown in Figure 17.

The PARAFAC model is symmetric in all modes and the number of components in each mode is the same [29]. The PARAFAC model is a simple model, which comes with a restriction of the equality on the number of components in each mode which makes it difficult to fit a data array with the PARAFAC model. One advantage of the PARAFAC model is its uniqueness: fitting the PARAFAC model with the same number of components to a given multiway dataset returns the same result.

#### Tucker3

**Figure 18:**
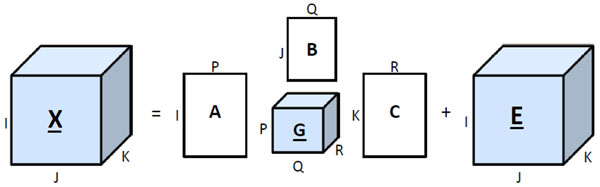
**Tucker3 model** Tucker3 model of a three-way array **X** with (*P*,*Q*,*R*) components at each mode. The tensor is decomposed into component matrices **A**, **B**, **C**, core array **G**, and residual array** E**.

Tucker3 is an extension of bilinear factor analysis to multiway datasets [30]. A 3-way array **X** ∈ ℝ*^I^*^×^*^J^*^×^*^K^* is modeled by a (*P*,*Q*,*R*)-component Tucker3 model as follows:

where **A** ∈ ℝ*^I^*^×^*^P^*, **B** ∈ ℝ*^J^*^×^*^Q^*, **C** ∈ ℝ*^K^*^×^*^R^* are the component matrices of first, second and third modes respectively. **G** ∈ ℝ*^P^*^×^*^Q^*^×^*^R^* is the core array and **E** ∈ ℝ*^I^*^×^*^J^*^×^*^K^* is the residual term. A description of the Tucker3 model is shown in Figure 18.

Tucker3 is a more flexible model compared to PARAFAC. This flexibility is due to the core array **G**, which allows interaction of any factor in a mode with any other factor in other modes [31]. Therefore, the number of components for each mode can be different. This results in indeterminacy of the Tucker3 model, since it cannot determine the component matrices uniquely.

**Model validation** A multiway model is appropriate if adding more components to any mode does not improve the fit considerably. There is a tradeoff between the complexity of the model and the variance of the data explained by the model. Therefore, validation of a model also determines a suitable complexity for the model. We used the core consistency diagnostic (CORCONDIA) to determine the number of components of the PARAFAC model [32]. The core consistency diagnostic measures the similarity of the core array **G** of the model and the superdiagonal array of ones. Core consistency is always less than or equal to 100% and may also be negative. As a rule of thumb, Bro et al. suggests that a core consistency above 90% implies a trilinear model [32]. In our experiments, we kept core consistency above 90%, while still explaining the variance of the data as much as possible with a trilinear model. We determined the number of components of the Tucker3 model by rank reduction on the unfolded tensor along each mode, and these components explain over 90% of the variance of the data.

#### Clustering algorithm

We developed the kmeans_mtimes_seeded algorithm, a modified version of the k-means algorithm, to group MTBC strains based on the score matrices of the multiway models. K-means is a commonly used clustering algorithm with two weaknesses: 1) Initial centroids are chosen randomly, 2) The objective value of k-means, measured as within-cluster sum of squares, may converge to local minima, rather than finding the global minimum. We solve these problems with two improvements: 1) Initial centroids are chosen by careful seeding, using a heuristic called kmeans++, suggested by Arthur et al. [33]. Let *D*(*x*) represent the shortest Euclidean distance from data point *x* to the closest center already chosen. kmeans++ chooses a new centroid at each step such that the new centroid is furthest from all chosen centroids. Algorithm 2 summarizes the kmeans++ procedure. 2) The local minima problem is partially solved by repeating the k-means algorithm multiple times and retrieving the run with the minimum objective value. We repeated the algorithm *m* = 20 times. The kmeans_mtimes_seeded algorithm combines these two improvements, as summarized in algorithm 3. The kmeans_mtimes seeded algorithm is more stable compared to the k-means algorithm, and produces more accurate clusters.

#### Cluster Validation

Clustering results for the MTBC strains are evaluated to determine the best choice for the number of clusters and compare the chosen clustering with existing sublineages using cluster validity indices. We used the best-match stability to pick the most stable clusterings. In case of a tie in average best-match stability, we used the DD-weighted gap statistic for cluster validation [34]. We compare our clusters to an existing classification using the F-measure.

**Best-Match Stability** The stability of a clustering is measured by the distribution of pairwise similarities between clusterings of subsamples of the data. The idea behind stability is that if we repeatedly sample data points and apply the same clustering algorithm to the subsample, then an effective clustering algorithm applied to well separated data should produce clusterings that do not vary much for different subsamples [35]. In such cases, the algorithm is stable independent of input randomization. We use best-match stability as suggested by Hopcroft et al. [36] to assess stability. The algorithm clusters the same data multiple times, and compares the reference cluster to model clusterings. We used 25 model clusterings to compare with the reference cluster. The stability of each cluster is calculated by finding the average best match between this cluster and the clusters identified using other model clusterings. High average best-match values denote that the two clusters have many strains in common and are of roughly the same size [8]. We also calculate the average best-match of a clustering by finding the average of best-match values for all clusters in the reference clustering. Best-match stability of a cluster C, compared to a model clustering , is calculated as:

where

and *refC_i_* is the set of items in reference cluster *i*.

**DD-Weighted Gap Statistic (PC)** Tibshirani et al. proposed a cluster validity index called the gap statistic, which is based on the within-cluster sum of squares (WCSS) of a clustering [37]. Let the dataset be *X* ∈ ℝ*^n^*^×^*^p^* consisting of *n* data points with *p* dimensions. Let *d_ij_* be the Euclidean distance between data points *i* and *j*. After clustering this dataset, suppose that we have *k* clusters *C*_1_, ‥, *C_k_*, where *C_i_* denotes the indices of data points in cluster *i*, of size *n_i_* =| *C_i_* |. The sum of within-cluster pairwise distances for cluster *r* is defined as:

and the within-cluster sum of squares for a clustering is defined as:

The idea of the gap statistic method is to compare *W_k_* and its expected value under a reference distribution of the dataset. Therefore, the gap value is defined as:

Where  represents the expected value under a sample of size *n* based on a reference distribution. The optimal number of clusters is the value  for which *Gap_n_*(*k*) is maximized. The selection of number of clusters via gap statistic is summarized in [37].

The reference distribution can be one of two choices: uniform distribution (Gap/Unif), or a uniform distribution over a box aligned with the principal components of the dataset (Gap/PC). Experiments by Tibshirani et al. show that Gap/PC finds the number of clusters more accurately, therefore we used Gap/PC in this study [37].

The gap statistic is a powerful method for estimating the number of clusters in a dataset. However, a study by Dudoit et al. showed that the gap statistic does not estimate the correct number of clusters for every case [38]. This may be because *W_k_* increases as the number of data points increases. Hierarchical structure of the data may also cause problems. The data may be composed of nested clusters and the gap statistic will be capturing only the minimum of these candidate numbers of clusters. Yan et al. suggested a 2-step improvement to the gap statistic, called the DD-weighted gap statistic [39]. They defined average within-cluster pairwise distances for cluster *r* as follows:

and the weighted within-cluster sum of squares  as:

Based on  ,the weighted gap statistic  is defined as

Let  denote the difference in  when the number of clusters is raised from k-1 to k.  is defined as

 for , and otherwise it will be close to zero. Therefore, to find a “knee” point in the plot, they introduce a second difference equation and define  as

 is maximized when k is equal to the true number of clusters. The advantage of  over the gap statistic is that there may be multiple peaks in the plot of  and this may indicate a hierarchical structure in the data. In such cases, multilayer analysis should be used instead of a single step procedure.

**F-measure** The F-measure is a weighted combination of precision and recall of a clustering. Since the F-measure combines precision and recall of clustering results, it has proven to be a successful metric. We use the F-measure to evaluate how similar the tensor sublineages are to the SpolDB4 families. According to the contingency table in Table 11, precision, recall, and F-measure are defined as:

**Table 11:** Contingency table

	Same cluster	Different clusters
Same class	a	b

Different classes	c	d

Contingency table of a clustering, where rows represent true classes and columns represent found clusters. Given *n* data points in the dataset, .

### Multiway Partial Least Squares Regression (N-PLS)

N-PLS is a multiway regression method where at least one of the independent and dependent blocks has at least three modes created by Bro et al. by generalizing PLS to multiway data [22]. Consider independent variables in the X-block, **X** ∈ ℝ *^I^*^×^*^J^*^×^*^K^*, and dependent variables in the Y-block, **Y** ∈ ℝ*^I^*^x^*^M^*. In our experiments, the X-block is a three-way array and the Y-block is a two-way array. The multiway array **X** is decomposed using a matricized version **X** ∈ ℝ *^I^*^×^*^JK^* as:

**X**=**t**(**w^K^** ⊗ **w^J^**)' + **E** (1)

and the two-way array **Y** is decomposed as:

**Y** = **uq'** + **F** (2)

where **t** ∈ ℝ*^I^*^×1^ and **u** ∈ ℝ*^I^*^×1^ are score vectors of **X** and **Y**. **w^J^** ∈ ℝ*^J^*^×1^ and **w^K^** ∈ ℝ*^K^*^×1^ are the loading vectors (weights) of the second and third modes of **X** respectively. q ∈ ℝ*^M^*^×1^ is the loading vector of **Y**. **E** ∈ ℝ*^I^*^x^*^JK^* and **F** ∈ ℝ*^I^*^x^*^M^* are the residuals of **X** and **Y** respectively.

Notice that the two-way array **Y** is decomposed into one score and one loading vector, whereas the matricized three-way array **X** is decomposed into one score and two loading vectors, **w^J^** and **w^K^**. This is the main difference between N-PLS and PLS. At each iteration of N-PLS, a new PLS component is added. If *n* PLS components are used, **X** is decomposed into component matrices **T** ∈ ℝ*^I^*^×^*^n^*, **W^J^** ∈ ℝ*^J^*^x^*^n^*, **W^K^** ∈ ℝ*^Kxn^*, and **Y** is decomposed into component matrices **U** ∈ ℝ*^I^*^×n^, **Q** ∈ ℝ*^M^*^×^*^n^*.

The aim of N-PLS is to maximize the covariance of **X** and **Y**. For this purpose, we define an inner relation linking the **X** and **Y** blocks, using their score matrices, **T** and **U**:

**U** = **TB** + **E_u_** (3)

This requires finding loading vectors **w^J^** and **w^K^** such that the covariance of **t** and **y** are maximized:

where **Z**∈ℝ*^J^*^×^*^K^* is a matrix with  and . To maximize this expression, we write it in matrix notation:

The problem of finding **w^J^** and **w^K^** is simply solved by SVD on **Z**[22,40]. **w^J^** and **w^K^** are first left and right singular vectors of **Z**. To reconstruct **Y**, we substitute (3) in equation 2:(4)

Given **X** and its decomposition matrices, we can make predictions for a new X-block, using equation 4. The derivation of the full and closed predictions with N-PLS has been presented by Smilde et al. [41]. Three alternative methods are proposed by De Jong et al. for derivation of training models via regression coefficients [42]. Bro et al. proposed an improved N-PLS method with better fit of the independent data, keeping regression coefficients and predictions the same [43].

The N-PLS model of a multiway array is a multilinear model, like PARAFAC, which means that it has no rotational freedom. Therefore, the N-PLS model of a multiway array is unique. In this study, we used a 3-way array as the X-block and a 2-way array as the Y-block, therefore we are particularly working on the Tri-PLS2 version of N-PLS, which is summarized in Algorithm 4. The term **X**_(1)_ in the algorithm refers to **X** matricized along the first mode. The X-block and Y-block are centered and scaled prior to application of the algorithm. The preprocessing and postprocessing of both X-block and Y-block are done according to centering and scaling methods explained in [44].

## Authors’ contributions

CO, KB and BY conceived the study. CO carried out the experiments. CO, KP and BY analyzed the results. AS and SV provided and analyzed some of the data. CO, AS, SV and KB drafted the manuscript.

## Competing interests

The authors declare that they have no competing interests.

## Supplementary Material

Additional file 1 **Confusion matrix of Euro-American strains** The confusion matrix of Euro-American strains that shows the correspondence of tensor sublineages and SpolDB4 families. Each row represents SpolDB4 families and each column represents tensor sublineages. Click here for file
